# The Spatiotemporal Expression of SOCS3 in the Brainstem and Spinal Cord of Amyotrophic Lateral Sclerosis Mice

**DOI:** 10.3390/brainsci14060564

**Published:** 2024-05-31

**Authors:** Ching-Yi Lin, Veronica Vanoverbeke, David Trent, Kathryn Willey, Yu-Shang Lee

**Affiliations:** Department of Neurosciences, Lerner Research Institute, Cleveland Clinic, LRI, NB3-90, 9500 Euclid Ave., Cleveland, OH 44195, USA

**Keywords:** amyotrophic lateral sclerosis (ALS), suppressor of cytokine signaling-3 (SOCS3), pre-Bötzinger Complex (preBötC), neuroinflammation

## Abstract

Amyotrophic lateral sclerosis (ALS) is characterized by the progressive loss of motor neurons from the brain and spinal cord. The excessive neuroinflammation is thought to be a common determinant of ALS. Suppressor of cytokine signaling-3 (SOCS3) is pathologically upregulated after injury/diseases to negatively regulate a broad range of cytokines/chemokines that mediate inflammation; however, the role that SOCS3 plays in ALS pathogenesis has not been explored. Here, we found that SOCS3 protein levels were significantly increased in the brainstem of the superoxide dismutase 1 (SOD1)-G93A ALS mice, which is negatively related to a progressive decline in motor function from the pre-symptomatic to the early symptomatic stage. Moreover, SOCS3 levels in both cervical and lumbar spinal cords of ALS mice were also significantly upregulated at the pre-symptomatic stage and became exacerbated at the early symptomatic stage. Concomitantly, astrocytes and microglia/macrophages were progressively increased and reactivated over time. In contrast, neurons were simultaneously lost in the brainstem and spinal cord examined over the course of disease progression. Collectively, SOCS3 was first found to be upregulated during ALS progression to directly relate to both increased astrogliosis and increased neuronal loss, indicating that SOCS3 could be explored to be as a potential therapeutic target of ALS.

## 1. Introduction

Amyotrophic lateral sclerosis (ALS, is often called Lou Gehrig’s disease) is a debilitating and devastating neurodegenerative disease characterized by the loss of both upper and lower motor neurons and ultimately paralysis and death, usually resulting from respiratory failure, within 2–5 years of diagnosis [1,2,3,4]. The majority of ALS cases are sporadic (sporadic ALS, i.e., sALS) with unknown etiology [5,6], whereas approximately 10% of cases correspond to inherited forms of ALS (familiar ALS, i.e., fALS). Mutations in the gene encoding for the enzyme Cu/Zn superoxide dismutase 1 (SOD1) are observed in approximately 20% of fALS cases [7]. ALS pathogenesis studied in ALS animal models are known to be attributable to oxidative stress, glutamate excitotoxicity, protein misfolding, mitochondrial defects, impaired axonal transport, or inflammation, any of which will eventually lead to motor neuron death. Despite the diverse etiologies of ALS [2,4], increasing evidence shows that progressive injury caused by excessive and extended neuroinflammation is a common determinant. Neuroinflammation is closely linked to the pathogenic mechanisms of acute or chronic neural injury in many diseases, and is regulated by a broad range of cytokines/chemokines [8,9,10,11]. A pivotal regulator of a broad range of cytokines/chemokines is Suppressor of cytokine signaling (SOCS). SOCS proteins negatively modulate signaling through the Janus kinase (JAK)/signal transducer and activator of transcription (STAT) pathway [12,13] to regulate neuronal growth and differentiation [14,15]. However, the role of SOCS in the CNS remains unclear.

Suppressors of cytokine signaling-3 (SOCS3), one member of the SOCS family of proteins, binds to gp130 (a common receptor for signal transduction with interleukin-6 (IL-6)), or JAK1 and JAK2, subsequently inhibiting signal transduction [16,17]. Expression of SOCS3 in neurons in particular causes a negative regulatory effect on signal transduction and transcription-3 (STAT3) activation, which consequently contribute to excitotoxic neuronal death and to decrease cell survival and neurite outgrowth in vitro [18,19,20,21,22]. In addition, SOCS3 negative regulation of neuronal survival and axon regeneration has been found both in vivo and in vitro [18,21,22,23,24]. SOCS3 is pathologically upregulated after neural injury/diseases to negatively regulate a broad range of cytokines/chemokines that mediate inflammation [13,25,26]; however, the spatiotemporal expression of SOCS3 in ALS has not been investigated previously.

In this study, we observed that SOCS3 levels are significantly upregulated in (a) the pre-Bötzinger Complex (preBötC) of the brainstem and (b) the ventral horn of both cervical and lumbar spinal cord of ALS mice, which are accompanied by increased astrogliosis and decreased neurons, and are related to the neurodegeneration stage of ALS from the pre-symptomatic to early symptomatic stage. Together, these findings demonstrate a potential role for the manipulation of SOCS3 levels to regulate ALS progression, which may uncover a promising and novel potential therapeutic target for balancing an uncontrolled inflammatory response.

## 2. Methods

### 2.1. Mice

All mouse experiments were performed in accordance with the protocols approved by the Institutional Animal Care and Use Committee at the Cleveland Clinic. The SOD1-G93A mouse, a transgenic mouse with a glycine (G)-to-alanine (A) conversion at the 93rd codon of the SOD1 gene in high copy number (SOD1-G93A) [27,28,29], is one of the most commonly used ALS models. SOD1-G93A (B6.Cg-Tg (SOD1*G93A)1Gur/J, Jackson Laboratory, JAX stock number 004435) on a C57BL/6J genetic background were the ALS mice model (ALS mice) used in the entire study. C57BL/6J mice (JAX stock No. 000664) were used as the WT mice for comparison.

All efforts were made to minimize animal suffering as well as the number of animals used. Male mice were used for the experiments throughout this study to exclude the possible impacts of sex difference on disease onset and lifespan [30]. All animals were housed in standard metallic mouse cages (19.5 × 29.5 × 15 cm) with a corncob bedding under standard conditions of constant temperature and controlled lighting (12/12 h light/dark cycle). Humidity was 55%, the temperature was 23 ± 1 °C, and food and water were available ad libitum. The rotarod and grip strength tests were performed during the light period. The animals were euthanized at either 9 weeks (pre-symptomatic stage) or 16 weeks (early symptomatic stage) of age. n = 8/group and then divided to n = 4/time point.

### 2.2. Rotarod Test

The rotarod test was used to assess motor function in a blinded fashion. Mice were trained on the rotarod twice at one week before recording the data. Beginning at 8 weeks old, all animals (n = 8/group) were weighed and evaluated for signs of a motor deficits using the accelerated rotarod test. For this test, the time for which an animal spent walking on the rotating rod of a rotarod apparatus (BX-ROD-M; Bioseb Instruments US, Pinellas Park, FL, USA) was recorded weekly. Each animal was given three tries. The longest latency (sec) it takes for the mouse to fall off the rod was recorded. The apparatus had an initial speed of 2 rpm, a ramp time of 300 s, and gradually accelerated at a rate of 38/300 rpm/s. The speeds therefore gradually and consistently increased from 2 rpm to 40 rpm over the course of 300 s. 

### 2.3. Grip Strength Test

The Grip strength test was also used to assess the motor function in a blinded fashion. During this test, each mouse was placed on the grip strength meter (BIO-GS4; BX-ROD-M; Bioseb Instruments US, FL, USA), n = 8/group. The tail was pulled gently to impel the mouse to grip the bar with its forelimbs, and then the tail was pulled backwards at a uniform speed until the mouse released its grip. Each animal was given three tries, and the maximal force (g) that the mouse released its grip was recorded as the grip strength.

### 2.4. Immunohistochemistry (IHC) Analyses 

After behavioral tests, ALS and control WT mice were terminated at either 9 or 16 weeks of age for IHC analyses in a blinded fashion. Animals anesthetized using Euthasol were transcardially perfused with ice-cold 0.9% saline followed by 4% paraformaldehyde (PFA). The brains and spinal cords were collected and post-fixed in 4% PFA for 1 day and were then transferred to 30% sucrose solution for 3 days. Frozen cryostat sections (30 μm thick each, 6~8 sections per animal) were incubated with phosphate-buffered saline (PBS) containing 0.3% Triton X-100 for 30 min at room temperature (RT), and then were incubated with the blocking solution (PBS containing 1% normal donkey serum (NDS, Vector Laboratories, Newark, CA), 3% bovine serum albumin (BSA, Sigma-Aldrich, St. Louis, MO, USA), and 0.5% Triton X-100 (Sigma-Aldrich, St. Louis, MO, USA)) for 60 min at RT and, subsequently, double-staining with SOCS3 antibody (rabbit, ab16030, 1:300, Abcam, Waltham, MA, USA) and either NeuN (mouse, MAB377, 1:500; EMD Millipore, Burlington, MA, USA), glial fibrillary acidic protein (GFAP) (mouse, MAB3402, 1:3000; EMD Millipore, Burlington, MA, USA), or ionized calcium-binding adaptor molecule 1 (Iba1) antibody (mouse, MA5-38265, 1:200; ThermoFisher Scientific, Waltham, MA, USA) at RT overnight. After washing in PBS containing 0.3% Triton X-100 three times, secondary donkey anti-rabbit IgG conjugated Alexa 555 (ab150074, 1:1200; Abcam, Waltham, MA, USA) and donkey anti-mouse IgG conjugated Alexa 488 (A-21202, 1:1200; ThermoFisher Scientific) were applied at RT for 2 h. After washing in PBS containing 0.3% Triton X-100 twice and then in PBS once, sections were covered with mounting medium containing Hoechest to stain nuclei (Vector Laboratories, Newark, CA, USA) and analyzed using a Zeiss confocal laser-scanning microscope. Steps were taken to minimize the potential for background staining by (1) application of both NDS and BSA during the incubation with blocking solution, primary antibodies, and secondary antibodies; and (2) conducting the regular staining procedures but with no addition of individual primary antibody against either SOCS3, NeuN, GFAP, or Iba1 to confirm the specificity of antibodies before IHC analyses.

### 2.5. Statistical Analyses 

Standardized areas for sampling in sections (IHC) from each animal in each group were selected using Photoshop and ImageJ. Light intensity and threshold values were maintained at constant levels for all analyses. The mean number of pixels containing immunoreactive product in the sampled area was measured and multiplied by the average intensity. This value was subtracted from background immunolabeled intensity, as measured in a separate adjacent section. Intensities were shown as mean ± standard error of mean (SEM) in units of the percentage of the maximal intensities from the individual animal, which are presented as 100%. Mean values for each animal were then normalized to obtain a percent intensity value for each group of mice. Statistical significance was evaluated using the two-tailed unpaired Student’s *t* tests for comparisons between groups. A one-way analysis of variance (ANOVA) followed by a Tukey test, or a two-way ANOVA followed by a Bonferroni test was used for multiple comparisons for IHC intensity. For behavioral assessments, statistical significance was evaluated using a two-way ANOVA followed by a Bonferroni test to determine whether significant differences existed between ALS and WT mice at different time points. Values of *p* < 0.05 were considered to be statistically significant, as evaluated using Graphpad Prism 8.4.3. The statistical analyses were performed in a double blinded fashion.

## 3. Results

### 3.1. Motor Function Is Declined in ALS Mice

**Figure 1:**
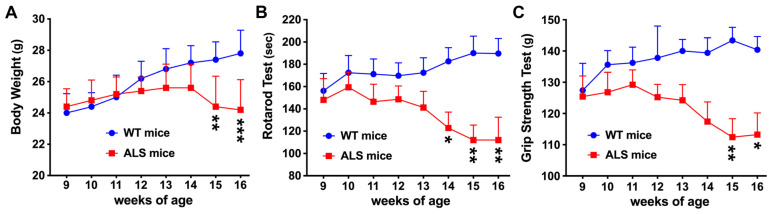
Progression of motor functional deficits in SOD1-G93A mice from 9 to 16 weeks of age. Body weight was measured every week (**A**). Motor function was assessed weekly in mice using the rotarod test (**B**) and Grip strength test (**C**). Data represent mean ± SEM. Statistical analysis was performed using a two-way ANOVA followed by a Bonferroni test (* *p* < 0.05, ** *p* < 0.01, and *** *p* < 0.001 vs. age-matched WT mice, n = 8 each group).

The ALS mice we used are SOD1-G93A mice (JAX stock No. 004435, Jackson Laboratories) which are on a C57BL/6J background with high copy number of transgene SOD1^G93A^ [27,28,29]. This is a well-characterized mouse model of ALS that develops initial signs of ALS around P100 (~ disease onset) [27,31,32,33] and reaches the end of life around P150 [31,33,34]. Disease onset was determined as the time when mice reached maximum body weight [35]. As shown in Figure 1A, ALS mice reached maximum body weight at 14 weeks old, and then gradually decreased their body weight after 15 weeks of age. Given that motor neurons are continuously lost over disease progression in the spinal cords of ALS mice, we used both rotarod and grip strength tests to assess the motor function of ALS mice as the disease progressed. The behavioral tests started at 9-week-old pre-symptomatic mice and ended at 16 weeks of age (early symptomatic stage). In the rotarod test, ALS mice showed a significant reduction in the time spent walking on the rod at 16 weeks of age compared with age-matched wild type (WT) mice (Figure 1B). The time the ALS mice spent on the rotarod was not significantly different at the pre-symptomatic stage (9 weeks of age), but was deteriorated by significantly decreased 77.6 s at the early symptomatic stage (16 weeks of age), as compared to the age-matched WT mice. In the grip strength test, there was no significant difference between ALS mice and WT mice at 9 weeks of age. However, ALS mice showed a significant reduction in grip strength by significantly decreased 27.2 g at 16 weeks of age when ALS progressed, as compared with age-matched WT mice (Figure 1C). Therefore, these data showed that motor dysfunction of ALS mice was progressively evident from pre-symptomatic to early symptomatic stage. We next investigated the spatiotemporal expression of SOCS3 in the relation to the progressive ALS pathology.

### 3.2. Increased Astrocytes and SOCS3 Upregulation Are Found in the Brainstem of ALS Mice

**Figure 2:**
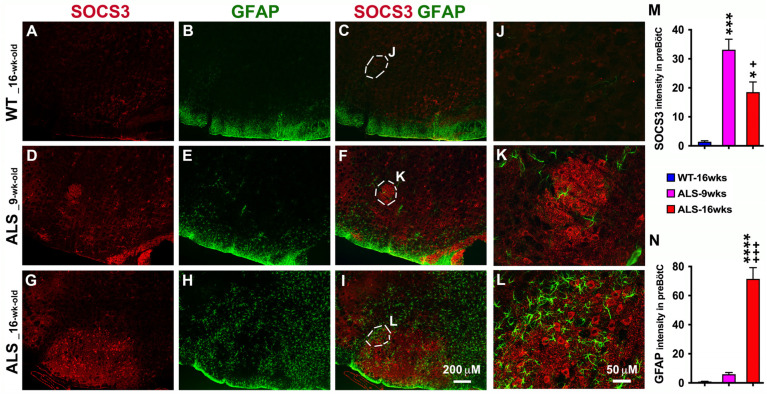
Upregulated SOCS3 in ALS mice is associated with increased astrocytes from the pre-symptomatic to early symptomatic stage in the brainstem. IHC was used to detect SOCS3 and GFAP+ astrocytes. As shown in the representative confocal images (**A**–**I**), significantly upregulated SOCS3 was observed in ALS mice at both 9 weeks of age (**D**,**F**) and 16 weeks of age (**G**,**I**) in the brainstem compared to WT mice (**A**,**C**). The levels of upregulated SOCS3 (**D**,**F**,**G**,**I**) were directly related with the extent of GFAP expression and astrocyte reactivity (**E**,**F**,**H**,**I**,**K**,**L**) over ALS progression. The ALS-induced increases in SOCS3 and GFAP+ astrocytes were especially significant in the preBötC of brainstem, as shown in the higher-magnified images (**K**,**L**), compared to WT mice (**J**). Graphs represent mean ± SEM of four animals per group per time-point for intensity of SOCS3 (**M**) and GFAP (**N**). * *p* < 0.05, *** *p* < 0.001, and **** *p* < 0.0001 vs. WT mice; + *p* < 0.05, and +++ *p* < 0.001 vs. 9-week-old ALS mice (one-way ANOVA followed by a Tukey test).

ALS is noteworthy for upper and lower motor neuron death, which eventually leads to muscle weakness and paralysis. The respiratory dysfunction and eventually respiratory failure especially accounts for a large portion of morbidity and mortality in ALS [36,37]. The brainstem is the structure which modulates breathing, heart function, and more, as well as containing pathways for communication between the brain and the spinal cord. We first used double fluorescent-staining to detect the levels of broad inflammation regulators, SOCS3 and GFAP-positive (GFAP+) astrocytes, in the brainstem of ALS mice. As shown in Figure 2, SOCS3 expression was significantly upregulated in the brainstem of ALS mice, especially in the preBötC of the brainstem (Figure 2D,F,G,I,K,L), compared to WT mice. The preBötC, a portion of the ventrolateral medullary reticular formation is thought to generate the inspiratory breathing rhythm in mammals [38,39]. In the preBötC, ALS-upregulated SOCS3 (Figure 2D,G,K,L) was associated with increased and reactive GFAP+ astrocytes (Figure 2E,H,K,L,N), indicating that the neuroinflammation already started before the disease onset as early as 9 weeks of age as examined, and kept ongoing after disease onset. However, there was no colocalization of SOCS3 and GFAP+ astrocytes (Figure 2F,I,K,L), which indicates that the resource of increased SOCS3 was not from GFAP+ astrocytes in the areas examined. 

### 3.3. ALS-Upregulated SOCS3 Associates with Increased Neuronal Loss in the Brainstem of ALS Mice

**Figure 3:**
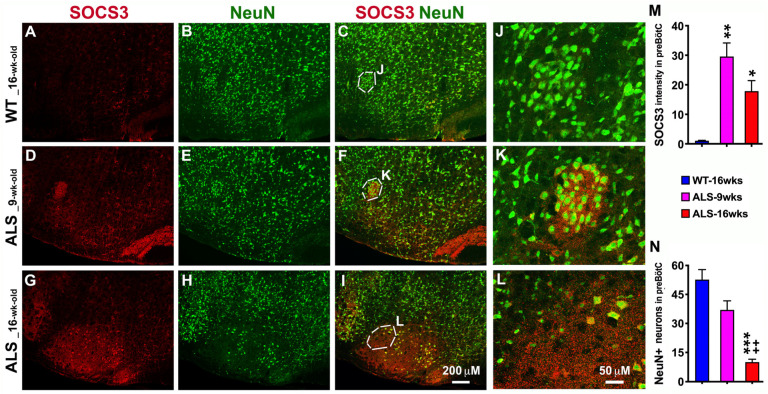
Upregulated SOCS3 in ALS mice during disease progression is directly associated with progressively increased neuronal loss in the brainstem. IHC was used to detect SOCS3 and NeuN+ neurons. As shown in the representative confocal images (**A**–**L**), significantly upregulated SOCS3 was observed in both 9-week-old (**D**,**F**) and 16-week-old (**G**,**I**) ALS mice in the brainstem, compared to WT mice (**A**,**C**). The levels of upregulated SOCS3 (**D**,**F**,**G**,**I**,**K**,**L**) were directly correlated with the extent of neuronal loss (**E**,**F**,**H**,**I**,**K**,**L**) over the course of ALS progression. Particularly, the ALS-in-duced increases in SOCS3 and NeuN+ neuronal loss were especially significant in the preBötC of brainstem, as shown in the higher-magnified images (**K**,**L**) compared to WT mice (**J**). Graphs repre-sent mean ± SEM of four animals per group per time-point for SOCS3 intensity (**M**) and NeuN+ neurons (**N**). * *p* < 0.05, ** *p* < 0.01, and *** *p* < 0.001 vs. WT mice; ++ *p* < 0.01 vs. 9-week-old ALS mice (one-way ANOVA followed by a Tukey test).

In addition, we used IHC to detect SOCS3 and NeuN+ (a marker for neurons) neurons in the brainstem of ALS mice and found that SOCS3 was significantly increased at the pre-symptomatic stage (9 weeks old, Figure 3D,F,K,M) and became more robust at the early symptomatic stage (16 weeks old, Figure 3G,I,L,M), when compared to WT mice. NeuN+ immunoreactivity was found in gray matter where neuronal cell bodies are located in both WT and ALS mice. Of most importance, the NeuN+ neurons were significantly decreased in the brainstem, especially in the preBötC of the brainstem in ALS mice at the pre-symptomatic stage (Figure 3E,F,K,N), and became even more exacerbated at the early symptomatic phases (Figure 3H,I,L,N), which was negatively related to the upregulated SOCS3 and was associated with neurodegeneration progression in a time-dependent manner. The NeuN+ neurons in the preBötC of the ALS mice were significantly decreased by 30 percent at 9 weeks of age, and were further decreased by 81 percent at 16 weeks of age, as compared to WT mice. Gradual and dramatic neuronal loss in the preBötC of the brainstem may account for the respiratory dysfunction over the ALS progression.

### 3.4. Significant Loss of Neurons When SOCS3 Is Pathologically Upregulated in the Cervical Spinal Cord of ALS Mice

**Figure 4:**
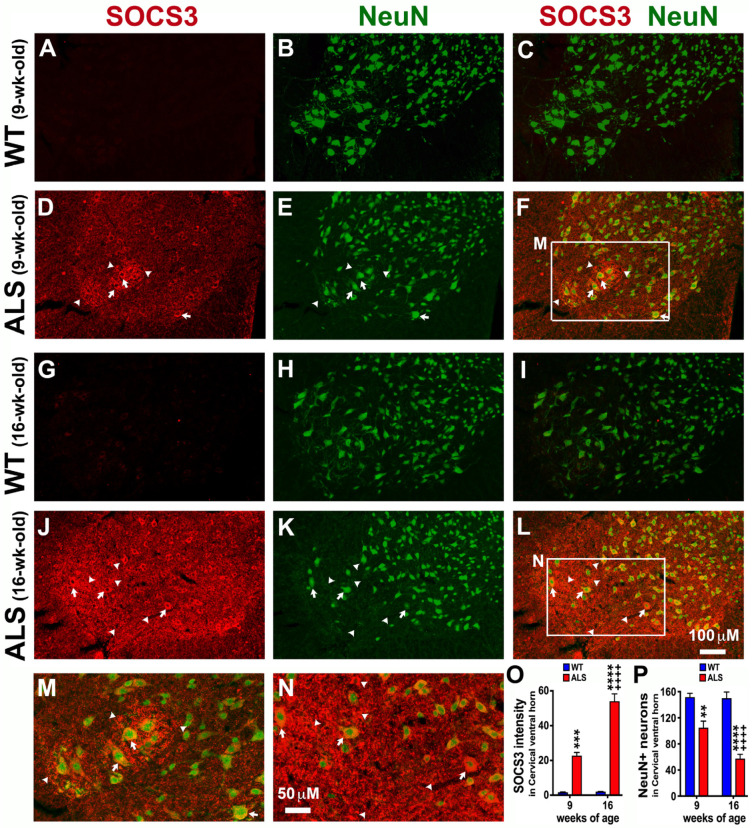
ALS significantly upregulates SOCS3 in the ventral horn of cervical spinal cord where neurons are lost. IHC was used to detect SOCS3 and NeuN+ neurons. Representative confocal im-ages show that SOCS3 levels were significantly upregulated in the ventral horn of the cervical spinal cord in ALS mice at 9 weeks (**D**,**F**) and 16 weeks (**J**,**L**) of age, as compared to WT mice at 9 weeks (**A**,**C**) and 16 weeks (**G**,**I**) old, respectively. Panels (**M**) and (**N**) are higher magnifications of the boxed areas (the major motor neuron pool in the ventral horn) in panels (**F**) and (**L**), respectively. The NeuN+ neurons were significantly decreased in ALS mice (**E**,**K**,**F**,**L**,**P**) over disease progression when compared to what found in WT mice (**B**,**H**,**C**,**I**,**P**). The ALS-increased SOCS3 was either co-localized with NeuN+ neurons (arrows) or not (arrowheads). Graphs represent mean ± SEM of four animals per group per time-point for SOCS3 intensity (**O**) and NeuN+ neurons (**P**). ** *p* < 0.01, *** *p* < 0.001, **** *p* < 0.0001 vs. age-matched WT mice; ++++ *p* < 0.0001 vs. 9-week-old ALS mice (two-way ANOVA followed by a Bonferroni test).

Severe motor neuron loss in the spinal cord is an important feature of ALS mice during disease progression [40]. Accordingly, we next examined the expression levels of SOCS3 in the spinal cords of ALS mice. To determine whether SOCS3 levels are increased in different level of spinal cord before and after the disease onset of ALS, we first examined the expression levels of SOCS3 in the cervical spinal cord at both the pre-symptomatic stage (9 weeks of age) and early symptomatic stage (16 weeks of age) of ALS mice using IHC analyses. As found in the brainstem (Figure 2 and Figure 3), IHC studies showed that the increased SOCS3 was largely located in NeuN+ neurons, especially the large neurons found in the ventral horn where motor neurons are mainly located (arrows in Figure 4F,L). SOCS3 was significantly increased by 12.5 times at 9 weeks of age (Figure 4D,F,M,O), and further increased by 25.3 times at 16 weeks of age (Figure 4J,L,N,O) in the cervical spinal cord of ALS mice, as compared to age-matched WT mice (Figure 4A,G,O). Moreover, not all increased SOCS3 was co-localized with NeuN+ neurons in the ventral horns of the cervical spinal cord; instead, some increased SOCS3 was co-localized with the traced fragment/debris or no signals of NeuN+ immunoreactivity (arrowheads, Figure 4F,L,M,N). These indicate that increased SOCS3 was produced by the dying or lost neurons, or even non-neuronal cells.

### 3.5. Significant Neuronal Loss and SOCS3 Upregulation Are Also Found in the Lumbar Spinal Cord of ALS Mice

**Figure 5:**
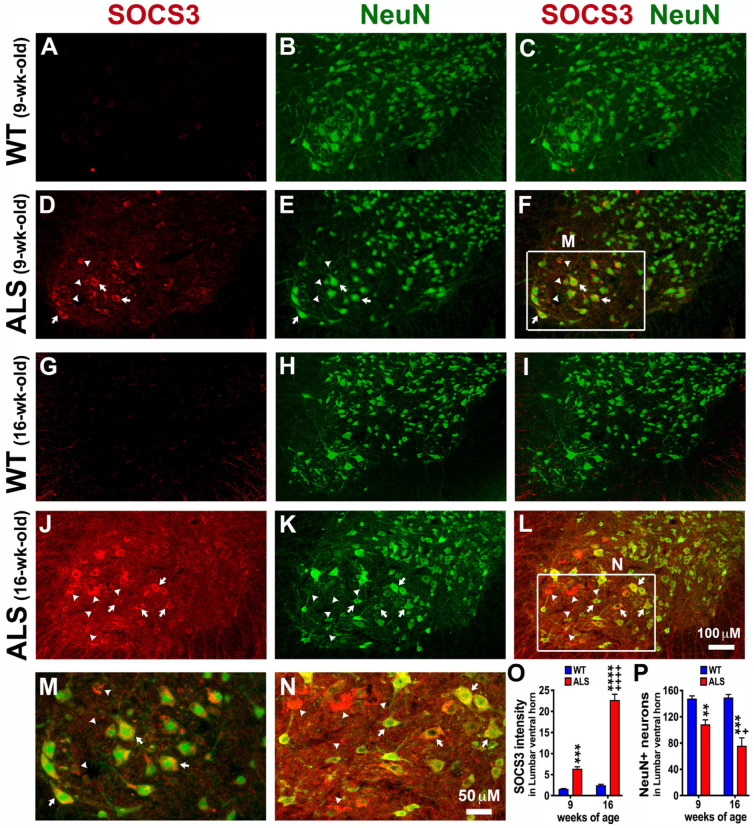
SOCS3 in SOD1-G93A mice is significantly upregulated in the ventral horn of the lumbar spinal cord where neurons are lost. IHC was used to detect SOCS3 and NeuN+ neurons. Representative confocal images showed that SOCS3 was significantly upregulated in the ventral horn of the lumbar spinal cord collected from ALS mice at both 9 (**D**,**F**) and 16 (**J**,**L**) weeks of age, as compared to the age-matched WT mice at 9 (**A**,**C**) and 16 weeks (**G**,**I**) old, respectively. In contrast, NeuN+ neurons were significantly decreased in ALS mice at both 9 (**E**,**F**) and 16 (**K**,**L**) weeks of age, as compared to the age-matched WT mice at 9 (**B**,**C**) and 16 weeks (**H**,**I**) old, respectively. Panels (**M**) and (**N**) are higher magnifications of the boxed areas (the major motor neuron pool in the ventral horn) in panels (**F**) and (**L**), respectively. The ALS-increased SOCS3 was either co-localized with NeuN+ neurons (arrows) or not (arrowheads). Graphs represent mean ± SEM of four animals per group per time-point for SOCS3 intensity (**O**) and NeuN+ neurons (**P**). ** *p* < 0.01, *** *p* < 0.001, and **** *p* < 0.0001 vs. age-matched WT mice; + *p* < 0.05 and ++++ *p* < 0.0001 vs. 9-week-old ALS mice (two-way ANOVA followed by a Bon-ferroni test).

Given that SOCS3 was highly upregulated in the neurons of the cervical spinal cords of ALS mice at the age of both 9 and 16 weeks (Figure 4), we next examined whether SOCS3 levels are upregulated in the lumbar spinal cords which modulates the hindlimbs function of ALS mice. Similar to what found in the cervical spinal cord (Figure 4), SOCS3 levels were found to be significantly upregulated at the pre-symptomatic stage (9 weeks of age, Figure 5D,F,M,O) and became exacerbated at the early symptomatic stage (16 weeks of age, Figure 5J,L,N,O) of ALS mice in the lumbar spinal cord, particularly in the large NeuN+ neurons found in the ventral horn where motor neurons are mainly located (arrows in Figure 5F,L–N). SOCS3 was significantly increased by 3 times at 9 weeks of age (Figure 5D,F,M,O), and increased by 8.2 times at 16 weeks of age (Figure 5J,L,N,O) in the lumbar spinal cord of ALS mice, as compared to age-matched WT mice (Figure 5A,G,O). The large NeuN+ neurons found in the ventral horn were significantly decreased by 26.5 percent at 9 weeks of age (Figure 5E,F,P) and decreased by 49 percent at 16 weeks of age (Figure 5K,L,P) in the lumbar spinal cord of ALS mice, compared to what we found in age-matched WT mice (Figure 5B,H,P). However, some increased SOCS3 immunoreactivity was not co-localized with NeuN+ neurons (arrowheads, Figure 5F,L–N), indicating that the increased SOCS3 was produced by not only NeuN+ neurons, but also the non-neuronal cells or the previous NeuN+ neurons, but died and were lost.

### 3.6. ALS-Upregulated SOCS3 Is Partially Co-Localized with Reactive Microglia/Macrophages in the Lumbar Spinal Cord

**Figure 6:**
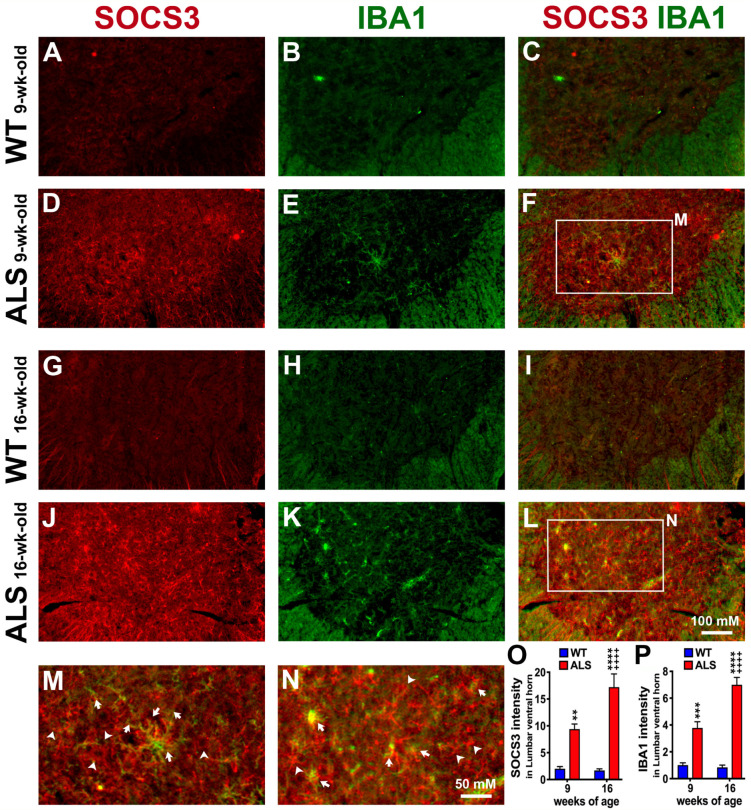
ALS-increased SOCS3 is partially co-localized with increased microglia/macrophages in the ventral horn of the lumbar spinal cord. IHC was used to detect SOCS3 and Iba1+ microglia/macrophages. Representative confocal images showed that SOCS3 was significantly increased in the ventral horn of the lumbar spinal cord collected from ALS mice at both 9 weeks (**D**,**F**) and 16 weeks (**J**,**L**) of age when compared to the age-matched WT mice at 9 weeks (**A**,**C**) and 16 weeks (**G**,**I**) old, respectively. In addition, Iba1+ microglia/macrophages were significantly increased in ALS mice (**E**,**K**,**F**,**L**,**P**) when compared to what found in WT mice (**B**,**H**,**C**,**I**,**P**). Panels (**M**) and (**N**) are higher magnifications of the boxed areas (the major motor neuron pool in the ventral horn) in panels (**F**) and (**L**), respectively. The ALS-increased SOCS3 was either co-localized with Iba1+ microglia/macrophages (arrows) or not (arrowheads). Graphs represent mean ± SEM of four animals per group per time-point for SOCS3 intensity (**O**) and Iba1+ neurons (**P**). ** *p* < 0.01, *** *p* < 0.001, and **** *p* < 0.0001 vs. age-matched WT mice; ++++ *p* < 0.0001 vs. 9-week-old ALS mice (two-way ANOVA followed by a Bonferroni test).

During ALS progression, significant microgliosis and astrocytic activation have been found in the spinal cord of ALS mice [41], which contribute to motor neuron degeneration, both in animal models [42,43] and in ALS patients [44]. Since we did not find the increased SOCS3 in astrocytes in the brainstem (Figure 2) and spinal cords. We next examined whether ALS-upregulated SOCS3 was found in microglia/macrophages using IHC analyses of SOCS3 and Iba1. Iba1 is a microglia/macrophage-specific calcium-binding protein [45]. Similar to the increases in SOCS3 levels, the Iba1+ microglia/macrophages were significantly increased by 2.7 times at the pre-symptomatic stage (9 weeks of age) (Figure 6E,F,P), and further increased by 7.3 times at the early symptomatic stage (16 weeks of age) (Figure 6K,L,P) in the lumbar spinal cord of ALS mice, as compared to age-matched WT mice (Figure 6B,H,P). Importantly, the increased SOCS was partially co-localized with Iba1+ microglia/macrophages at 9 weeks of age (arrows in Figure 6F,M), and became more evident at 16 weeks of age (arrows in Figure 6L,N).

## 4. Discussion

In this study, we are the first to explore the potential role of SOCS3 in neuroinflammation and neuronal loss in ALS by examining the spatiotemporal expression of SOCS3 in SOD1-G93A ALS mice. IHC analyses showed that SOCS3 protein levels were significantly increased in both the ventral horn of spinal cords and preBötC of the brainstem of ALS mice at the pre-symptomatic stage; such increases were exacerbated at the early symptomatic phase. Concomitantly, SOCS3 levels were significantly upregulated over the course of the disease progression, and were directly related to the increased reactive astrogliosis and significant neuronal loss. Furthermore, SOCS3 levels were significantly upregulated prior to the disease onset, which suggest that SOCS3 may play a role in preceding ALS progression by the regulation of neuroinflammation.

The SOD1-G93A mice we have used are B6.Cg-Tg (SOD1*G93A)1Gur/J which have a high copy number of transgene SOD1^G93A^ to have quicker disease onset and shorter lifespan, as compared to another SOD1-G93A mice with a low copy number of transgene SOD1^G93A^. While there is no universal rule to define the disease onset due to individual variability and disease complication, we have chosen to follow the previous study [35] to determine disease onset as the time when mice reached maximum body weight; it is thus 14 weeks old in this study, as shown in Figure 1. To study if there are dynamic changes in SOCS3 levels as the disease progressed and to avoid the influence of variable disease onset in the SOD1-G93A mice, we have used 9-week-old mice for the pre-symptomatic studies and 16-week-old mice for the early symptomatic studies. The two time points allow us to detect the clear changes in the motor function, SOCS3 levels, astrogliosis, and neuronal loss, which provide critical insights into the dynamic SOCS3 levels as ALS progressed and a reasonable rationale to further investigate the SOCS3 levels in the late symptomatic stage and even the disease endpoint in the future.

An important feature of ALS mice during disease progression is severe motor neuron death that leads to progressive muscle wasting and paralysis [40]. The behavioral performance declined, as shown in Figure 1, and the motor function of ALS mice went into deficit after disease onset. Motor neurons typically have several dendrites and a large cell body which is located in the motor cortex, brainstem, or spinal cord. To study the full neuron profiles involved in the SOCS3 expression, we have chosen an antibody specific to NeuN rather than the motor neuron marker Choline Acetyltransferase (ChAT) for this study. While NeuN is a general neuronal marker, a portion of NeuN+ neurons with increased SOCS3 levels are known to be motor neurons based on their size and location in preBötC of the brainstem and ventral horn of the spinal cord. Importantly, our data show that not only motor neurons, but also another type of neurons are dying or lost with the increases in SOCS3 over ALS progression. Future studies will be needed to use different antibodies, including ChAT, to prove their identity. In addition, the expression of SOCS3 is not found to be different between WT and ALS mice in the cerebral pyramidal cells, which is relatively low, as compared to what we found in the brainstem and spinal cord.

We are interested in investigating SOCS3 expression in the brainstem, as most ALS patients are dying of respiratory complications, while their clinical presentations can be different. We find that SOCS3 levels were significantly increased from the pre-symptomatic to early symptomatic stage in the brainstem, especially in the preBötC. In parallel, we find that the neurons in preBötC of the brainstem were significantly lost over the course of ALS progression, which supports the evidence that dysfunction in the central control of breathing in some ALS patients may be related to preBötC degeneration [36]. Our findings suggest that respiratory neuron pools for breathing control are directly involved in progressive ALS pathology at the very early stage, and challenge the traditional explanation of respiratory failure in ALS, being that it causes motor neuron degeneration resulting in a weakness of the diaphragm and upper airway muscles, compromising the pump and patency of ventilation [46]. While we find that SOCS3 expression is significantly increased in the preBötC of ALS mice based on its unique anatomic location, future studies incorporating specific markers, such as Neurokinin-1 Receptor (NK1R), Somatostatin (SST), ChAT, and Paired-Like Homeobox 2B (PHOX2B), are needed to more accurately distinguish the preBötC from Nucleus Ambiguus.

Neuroinflammation has been found in both ALS autopsy cases and experimental mouse models, and is a prominent pathological feature that is commonly found at sites of motor neuron injury [47]. Although it is not yet fully understood, an inflammatory process inside the central nervous system (CNS), including pro-inflammatory cytokines, has been considered to contribute to the pathogenesis of ALS [48,49]. It has been postulated that in the earliest stage of ALS, motor neurons release signals to trigger microglia responding in an anti-inflammatory phenotype to release neuroprotective factors in an attempt to repair motor neurons and to protect them from further injury. However, as the disease progress, motor neurons release signals that transform microglia from a protective anti-inflammatory to a cytotoxic pro-inflammatory phenotype, which causes astrocytic dysfunction, astrogliosis, and enhances motor neuron degeneration. While the cause for this phenotypic variability remains unknown; cytokines appear to be likely candidates for such changes [47,49]. Although definitive involvement of the SOCS family of proteins in ALS has not yet been directly explored, SOCS3, a cytokine-inducible protein, has been shown to be required for pro-inflammatory macrophage activation in vivo [50]. Without SOCS3, macrophages develop characteristic anti-inflammatory markers, even when exposed to pro-inflammatory stimuli [50]. These results are consistent with the findings that pro-inflammatory macrophages have been associated with increased SOCS3 [51]. This is also consistent with reports that mice with conditionally deleted SOCS3 expression in nestin-expressing cells have increased STAT3 activation, thereby limiting infiltration of inflammatory cells and subsequent neuron and oligodendrocyte death, which in turn leads to improved functional recovery after spinal cord injury (SCI) [12,52]. 

It is important to note, however, that although SOCS3 is a member of the SOCS protein family, which was initially characterized by its negative regulatory effects on cytokine signaling, the prevailing and most well-studied function of SOCS3 is to inhibit JAK/STAT signaling. Additional signaling pathways, such as the mitogen-activated protein kinases (MAPK), nuclear factor kappa-light-chain-enhancer of activated B cells (NF-kB) pathways, and insulin signaling are also modulated by SOCS3 [50], as shown in the acute injury models. The situation in a primary neurodegenerative disease may be quite different, and the consensus is that glial (inflammatory) responses drive disease progression in the SOD1-G93A ALS mice [53]. The SOCS3 upregulation found in ALS mice reflects ongoing and chronic neuroinflammation in the nervous system of ALS mice. More experiments will be needed to measure cytokine release and STAT3 activation in order to fully understand the whole picture on how SOCS3 influences the inflammatory responses, which may be the primary driver of ALS. The previous research findings for SOCS3 so far are either supportive or contradictory to each other, which could be due to the complexity of the mechanisms that SOCS3 regulates and the diversity of targets/signaling pathways involved. Additional experiments are therefore required to explore the roles that SOCS3 plays in response to a variety of both physiological and pathological conditions. Therefore, in the case of ALS, it appears evident that it would be extremely useful to investigate the roles of SOCS3, especially in order to develop new therapeutic approaches.

Surprisingly, SOCS3 protein levels were already significantly increased at the pre-symptomatic stage as early as 9 weeks old as examined, indicating that the ongoing neuroinflammation already started during the preclinical phase of the disease and preceded the ALS progression. These preclinical effects may be considered to be critical parameters for the future design of pharmacological trials in ALS. Most importantly, in this study, we are the first to report that increased SOCS3 levels (1) are directly associated with the increases in reactive astrocytes and microglia/macrophages in the ALS mice, and (2) are directly associated with the increases in the neuronal loss over the course of disease progression as early as at the pre-symptomatic stage and become more exaggerated in the early symptomatic stage. These findings support the hypothesis that microglia/macrophages-induced non-cell-autonomous toxicity on neurons is involved in the progressive ALS pathology [35,53,54] and call for further preclinical investigations to determine if SOCS3 plays a role in the pathogenesis of ALS, especially in the relation to the motor neuronal death via the non-cell-autonomous pathway.

## 5. Conclusions

The studies of the spatiotemporal expression of SOCS3 in ALS not only suggest the involvement of the neuroinflammation-associated non-cell-autonomous pathway in the progressive ALS pathogenesis, but also provide a potential therapeutic target for balancing an uncontrolled neuroinflammatory response through the manipulation of SOCS3 levels to regulate ALS progression.

## Data Availability

Data are contained within the articles.
